# Clinical associations of IL-10 and IL-37 in systemic lupus erythematosus

**DOI:** 10.1038/srep34604

**Published:** 2016-10-06

**Authors:** Jack Godsell, Ina Rudloff, Rangi Kandane-Rathnayake, Alberta Hoi, Marcel F. Nold, Eric F. Morand, James Harris

**Affiliations:** 1 Rheumatology Group, Centre for Inflammatory Diseases, School of Clinical Sciences at Monash Health, Faculty of Medicine, Nursing & Health Sciences, Monash University, Clayton, Victoria, Australia; 2 Ritchie Centre, Hudson Institute of Medical Research, Clayton, Victoria, Australia; 3 Department of Paediatrics, School of Clinical Sciences at Monash Health, Faculty of Medicine, Nursing & Health Sciences, Monash University, Clayton, Victoria, Australia

## Abstract

Systemic lupus erythematosus (SLE) is a systemic autoimmune disease characterized by the development of autoantibodies to nuclear antigens and inflammatory responses mediated by multiple cytokines. Although previous studies have determined clinical associations between SLE and the anti-inflammatory cytokines IL-10 and IL-37, their role in the disease, or their potential as biomarkers, remains unclear. We examined serum levels of IL-10 and IL-37 in a large cohort of SLE patients, with detailed longitudinal clinical data. We demonstrate a statistically significant association of serum IL-10 with disease activity, with higher levels in active compared to inactive disease. High first visit IL-10 was predictive of high subsequent disease activity; patients with IL-10 in highest quartile at first visit were 3.6 times more likely to have active disease in subsequent visits. Serum IL-37 was also higher in SLE patients compared to control, and was strongly associated with Asian ethnicity. However, IL-37 was not statistically significantly associated with disease activity. IL-37 was significantly reduced in patients with organ damage but this association was attenuated in multivariable analysis. The data suggest that IL-10, but not IL-37, may have potential as a biomarker predictive for disease activity in SLE.

Interleukin-10 (IL-10) is a homodimeric protein that is produced by macrophages, dendritic cells and helper T cells in response to multiple stimuli^1^,^2^,^3^. Actions of IL-10 on immune cells *in vitro* include the inhibition of both macrophage and T cell activation and of pro-inflammatory cytokine production^4^,^5^,^6^,^7^,^8^,^9^. In contrast, IL-10 increases B cell survival, proliferation, differentiation and antibody production^10^, and decreases auto-reactive B-cell apoptosis by increasing *Bcl-2* expression, resulting in increased autoantibody production in systemic lupus erythematosus (SLE)^11^,^12^. Enhanced production of IL-10 has been reported in SLE and systemic sclerosis, while deficiency of IL-10 is reported in inflammatory bowel disease, psoriasis and rheumatoid arthritis^1^.

Previous studies evaluating clinical associations of IL-10 in SLE have mostly been small, retrospective, or have had other methodological limitations. In these smaller studies, serum concentrations of IL-10 have consistently been shown to be higher in SLE patients compared with healthy controls, and to positively correlate with measures of disease activity^13^,^14^,^15^, but the predictive value of IL-10 as a biomarker has not been examined in longitudinal studies. One study of administration of an anti-IL-10 monoclonal antibody in patients with SLE demonstrated a reduction in disease activity, consistent with a pathogenic role for IL-10 in SLE, but this finding is limited by the small sample size and lack of a control group^16^. In mouse models of SLE, IL-10 neutralization effectively delayed disease onset, but IL-10 deficiency resulted in more severe disease earlier in the disease course^17^. In contrast, IL-10 overexpression delayed the onset of autoantibody production and decreased nephritis in murine lupus^19^, suggesting that IL-10 can be either protective or pathologic at different stages of the disease^20^. Indeed, as the pathogenic drive in SLE shifts from innate immunity to a predominantly B-cell centric model, so too might the role of IL-10^21^.

Like IL-10, IL-37 is a cytokine known to have anti-inflammatory effects, but whose range of actions relevant to SLE are poorly understood. A member of the IL-1 family, IL-37 was first identified in 2000 by three independent groups^22^,^23^,^24^ as a new molecule within the IL-1 family locus on chromosome 2q13. Initially referred to as IL-1 family member 7 (IL-1F7), it was renamed IL-37 in 2010, as its unique physiological functions came to light^25^,^26^.

Constitutive levels of *IL1F7* (the gene encoding IL-37) mRNA are low in human monocytes in the absence of inflammatory stimulation^27^, while TLR ligands such as LPS up-regulate *IL1F7* transcription and increase *IL1F7* mRNA stability^28^. Like IL-1β and IL-18, IL-37 is produced as a pro-form, which can undergo processing by caspase-1. IL-37 is expressed as multiple splice variants, with the longest and likely the biologically most relevant isoform, IL-37b, being expressed in monocytes and lymph nodes^28^,^29^. IL-37 uses a heterodimeric receptor complex comprising IL-1 receptor 8 (IL-1R8) and the alpha chain of the IL-18 receptor (IL-18Rα) to activate an intracellular anti-inflammatory program, inhibiting the kinases Fyn and TAK1, as well as mitogen-activated protein kinases (MAPK) and the transcription factor NF-κB^30^.

Only one previous study has examined the expression of IL-37 in SLE. Song *et al*. measured IL-37 levels in the sera of 30 patients with SLE and demonstrated a strong positive correlation between IL-37 levels and disease activity, as measured using the SLE disease activity index 2000 (SLEDAI-2k)^31^. Correlation between *IL1F7* mRNA expression and mRNA of other inflammatory cytokines, including *IL18*, *IL6* and *IFNG*, in SLE peripheral blood mononuclear cells (PBMCs) was also shown.

As limited studies have informed the value of serum IL-10 and IL-37 as biomarkers of SLE disease state, their potential as therapeutic targets remains poorly understood. Therefore, we undertook a study of clinical associations of these two anti-inflammatory cytokines in a large cohort of patients with SLE with detailed longitudinal clinical information.

## Results

### Patient Characteristics

Cytokines were quantified in a total of 141 serum samples obtained at a first study visit, and in 251 additional samples obtained from a subset of 129 patients at consecutive subsequent clinic visits within an approximately 24-month period. Patients’ characterisitcs are presented in Table 1. In brief, 84% of the patients were females, with a median age of 42 years and 41% (n = 58) were of Asian ethnicity (Table 1). The median age at first visit was 42 (IQR 32, 52) years and median disease duration was 103 (18–439) months. At first visit, 55 patients (39%) were found to have active disease (SLEDAI-2k > 4); of these, 17 patients had highly active disease (SLEDAI-2k ≥ 10). Medications used by the patients during study period are shown in Table 1; approximately 66% (n = 93) of patients were taking prednisolone (PNL) at the first visit of this study.

### IL-10 and IL-37 concentration in SLE vs. Controls

**Figure 1:**
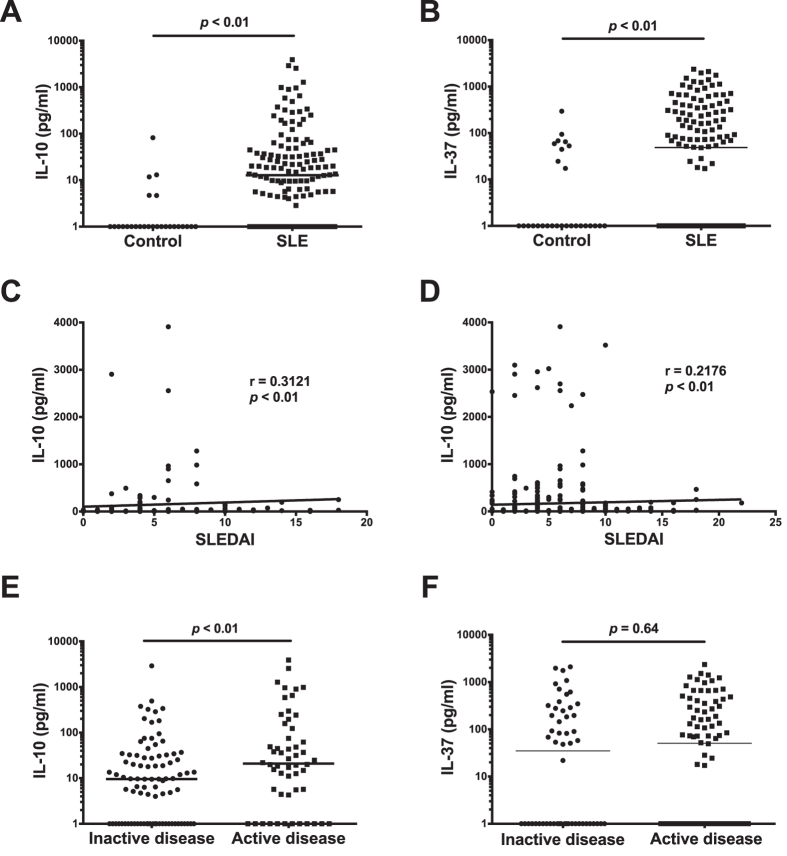
Serum IL-10 is increased in SLE and active disease. Serum levels of (**A**) IL-10 and (**B**) IL-37, measured by ELISA, in patients with SLE, compared with healthy controls. Correlations graphs of (**C**) First visit IL-10 and (**D**) IL-10 from all visits with SLEDAI-2k. (**E**) Comparison of Serum IL-10 in patients with inactive (SLEDAI-2k < 4) or active disease (SLEDAI-2k > 4) (**F**) Comparison of serum IL-37 levels in patients with inactive or active disease. The horizontal line in each dot plot depicts the median.

IL-10 was detectable in 128 SLE patient samples (90.1%) at first visit and 91.8% of all samples. IL-10 measured at first visit was found to be significantly higher in the serum of SLE patients compared to controls (*p* < 0.01, Fig. 1A). This significant difference was reproduced at each individual time point, and when all results were pooled (data not shown).

IL-37 was detectable in 77 SLE samples (54.2%) at first visit and 64.3% of all samples. At first visit, IL-37 was statistically significantly higher in SLE patients compared with controls (*p* < 0.01) (Fig. 1B). The finding of significantly higher serum IL-37 in SLE patients compared to controls was reproduced at each individual time point, and when all results were pooled (data not shown).

### IL-10, IL-37 and SLE disease activity

We examined correlations between IL-10 and IL-37 and disease activity. A modest but statistically significant positive correlation was observed between first visit IL-10 and SLEDAI-2k (r = 0.3121, *p* < 0.01) (Fig. 1C). This correlation was reproduced when data from all visits was examined (r = 0.2176, *p* < 0.01) (Fig. 1D). When patients were categorized as having inactive (SLEDAI-2k ≤ 4) or active (SLEDAI-2k > 4) disease, a statistically significantly higher median IL-10 concentration was observed in those with active disease (*p* < 0.01) (Fig. 1E).

In contrast, in the same samples, no significant correlations were found between IL-37 and disease activity (r = −0.2910, *p* = 0.7) (not shown). No significant difference in median IL-37 was detected between patients categorized as inactive (SLEDAI-2k ≤4) or active (SLEDAI-2k > 4) (p = 0.6) (Fig. 1F).

### Factors associated with serum IL-10

We next examined several factors associated with IL-10 levels measured at first visit, including demographics, disease activity, organ damage and PNL use, using linear regression analysis. Univariable analysis revealed Asian ethnicity, PNL use and disease activity (SLEDAI-2k > 4) were associated with elevated IL-10 expression with statistical significance (Table 2). Patients with Asian ethnicity had more than twice the amount of serum IL-10 when compared to non-Asians (ratio of the GM = 2.2 [95% CI 1.09, 4.42]; *p* = 0.03). Similarly, patients on PNL at first visit also had significantly higher IL-10 (ratio of the GM = 2.6 (95% CI 1.25, 5.31; *p* = 0.03). Patients with active disease had serum IL-10 concentrations nearly three times higher than those with inactive disease. There were no associations between the use of other immunomodulatory medications, including hydroxychloroquine, methotrexate, azathioprine and mycophenolate mofetil, with IL-10 expression.

In the multivariable linear regression analysis, only disease activity remained statistically significantly associated with serum IL-10 (Table 2). Interestingly, the association between PNL use and IL-10 disappeared when adjusted for disease activity; meaning disease activity confounded the association between PNL and IL-10. The association between Asian ethnicity and increased IL-10 was also attenuated (Table 2). Use of other drugs (Table 1) was too variable among patients to be the subject of analysis for associations with serum IL-10.

### Factors associated with serum IL-37

Similarly, factors associated with serum IL-37 measured at first visit were examined and the results are presented in Table 3. Again, Asian ethnicity was statistically significantly associated with increased serum IL-37 levels when compared to non-Asians, and this association remained statistically significant in multivariable linear regression analysis (adjusted ratio of the GM = 3.58 [95% CI 1.34, 9.58]; *p* = 0.01). In contrast, disease activity was not found to be associated with IL-37 expression. In addition, we observed significantly reduced levels of IL-37 in patients who had organ damage at first visit during univariable analysis but the statistical significance was attenuated when adjusted for ethnicity (adjusted ratio of the GM = 0.42 [95% CI 0.17, 1.07]; *p* = 0.07).

### First visit IL-10 levels and subsequent disease activity

We further examined whether levels of IL-10 measured at the first visit could predict subsequent disease activity. This analysis was restricted to patients with two or more visits; multivariable logistic regression analysis was carried out and the results are summarised in Table 4. Patients whose IL-10 levels were in the highest quartile were 3.6 times more likely to experience active disease (SLEDAI-2K > 4) even after adjusting for prednisolone use and ethnicity (adjusted odds ratio = 3.63 [95% CI; 1.31, 10.1], *p* = 0.01). PNL use at the first visit was also a predictor of subsequent active disease (Table 4).

In addition to the association with overall disease activity, a significant positive correlation was seen between IL-10 measured at the first visit and commonly used markers of inflammation in SLE, including erythrocyte sedimentation rate (ESR) (r = 0.2189, *p* < 0.01) and anti-dsDNA antibodies (r = 0.2175, *p* < 0.01), and corresponding significant negative correlations with C3 (r = −0.2360, *p* < 0.01) and C4 (r = −0.2023, *p* = 0.02) (not shown). Similar statistically significant correlations between serum IL-10 and ESR, dsDNA, C3 and C4 were observed in the samples taken at each of the subsequent study timepoints (data not shown). No significant correlations were found between IL-37 and ESR, anti-dsDNA antibody, C3 and C4.

**Figure 2:**
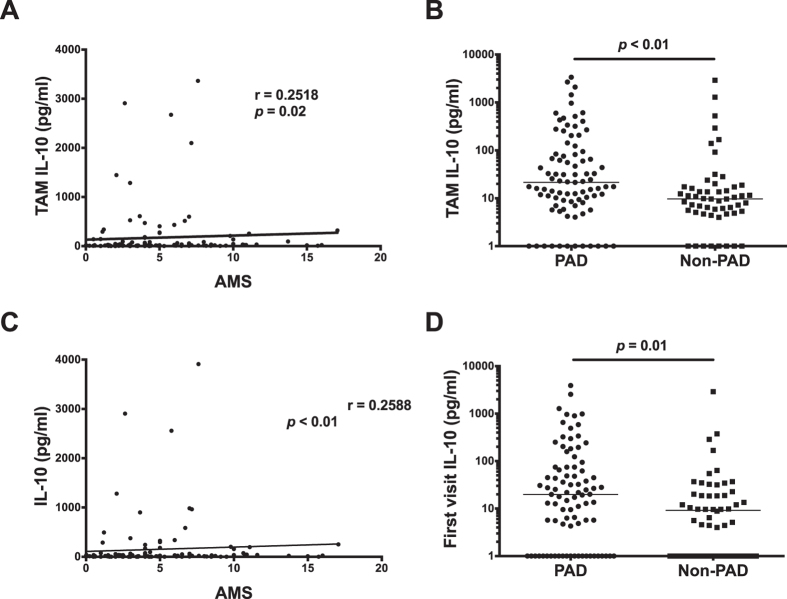
Serum IL-10 is correlated with longitudinal assessments of disease activity in SLE. (**A**) Correlation between time-adjusted mean (TAM) serum IL-10 and time adjusted mean SLEDAI-2k (AMS). (**B**) comparison of TAM IL-10 in serum from patients with or without persistently active disease (PAD), measured by ELISA. (**C**) Correlation between first visit serum IL-10 with AMS. (**D**) Comparison of first visit IL-10 in serum from patients with or without persistently active disease (PAD), measured by ELISA. The horizontal line in each dot plot depicts the median.

We also assessed the relationship between IL-10 and disease activity when both were measured over time, using the time-adjusted mean SLEDAI-2k (AMS) and time-adjusted mean (TAM) IL-10. TAM IL-10 was statistically significantly correlated with AMS (r = 0.2518, *p* = 0.02) (Fig. 2A). TAM IL-10 was also found to be higher in patients who suffered an episode of persistently active disease (PAD) during their disease course, compared to those who did not (*p* < 0.01) (Fig. 2B). IL-10 measured at the first visit was also significantly correlated with AMS (r = 0.2588, *p* < 0.01) (Fig. 2C) and was significantly higher in patients with subsequent PAD (*p* = 0.01) (Fig. 2D). In contrast, no statistically significant correlation was demonstrated between first visit IL-37 and subsequent disease activity (Supplementary Figure S1).

### IL-10, IL-37 and organ involvement

**Figure 3:**
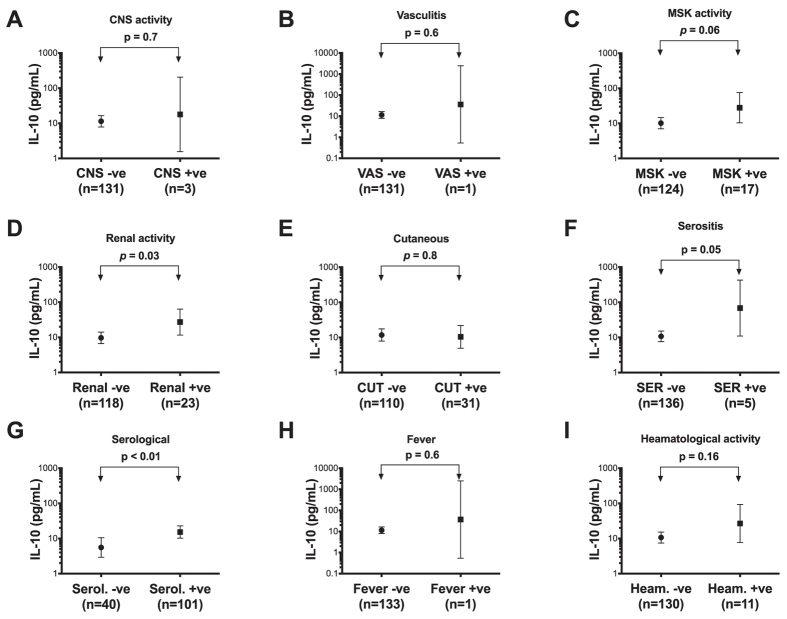
Associations of serum IL-10 with organ-specific disease activity in SLE. Geometric mean concentrations of IL-10 (with corresponding 95% confidnece interval in pg/mL), derived from univariable linear regression analyses by organ-specific disease; (**A**) Central nervous system (CNS) activity, (**B**) vasculitis (VAS), (**C**) musculoskeletal (MSK) activity, (**D**) renal activity, (**E**) Cutaneous (CUT) disease, (**F**) Serositis (SER), (**G**) Serological (Serol.) activity, (**H**) Fever, (**I**) Heamatological (Heam.) activity. Values are geometric means.

We next examined the association between IL-10 and IL-37 and specific organ involvement using the organ-domain SLEDAI-2k scores. Musculoskeletal activity (*p* = 0.05), renal activity (*p* = 0.03), serositis (*p* = 0.05) and serological activity (*p* < 0.01) were each found to be associated with increased IL-10 expression (Fig. 3). No significant difference in serum IL-10 was seen between SLE patients with and without CNS, vasculitis or cutaneous disease or in patients with or without fever or heamatoligical activity (Fig. 3).

## Discussion

Many cytokines are raised in the serum of SLE patients, reflective of the systemic inflammation inherent in the disease^32^. Previous studies have demonstrated that IL-10 is increased in the serum of SLE patients compared to controls^13^,^14^,^15^,^33^,^34^,^35^,^36^,^37^,^38^,^39^. Despite the uncertainty regarding its function, the consistency with which higher IL-10 levels are seen is informative about the altered cytokine milieu in SLE, particularly as studies, including this one, have shown a positive association between IL-10 and disease activity. In the current study we show that at an individual time point, and also when integrated over time, elevated serum IL-10 is associated with overall disease activity as measured by SLEDAI-2k.

The association observed between IL-10 and disease activity is supported by the correlations seen between IL-10 and other markers of disease severity, such as active renal disease and patients’ ESR, C3 and C4. As these laboratory markers are commonly used in addition to clinical features as indicators of disease activity, their correlation with IL-10 supports its potential as a marker of disease activity. Serum IL-10 has previously been found to be higher in patients with active nephritis compared to patients with inactive disease or controls, although interpretation of that study was limited by a sample size of 12^40^. In addition, levels of serum IL-10 correlated with anti-dsDNA antibody titers, as has been previously reported^13^,^14^,^15^. This correlation highlights a potential mechanism by which IL-10 might contribute to pathology in established SLE. The ability of IL-10 to enhance B-cell survival, proliferation, differentiation and antibody production, as well inhibiting auto-reactive B-cell apoptosis, may contribute to elevated anti-dsDNA titers in SLE patients^10^,^11^,^12^. Given that circulating immune complexes have been shown to increase IL-10 synthesis and that IL-10 can, in turn, facilitate the production of autoantibodies, it could be suggested that IL-10 acts pathogenically in SLE by amplifying and perpetuating an inflammatory cycle^1^,^2^,^41^. This hypothesis requires further *in vitro* and *in vivo* exploration.

IL-10 has only been measured in a longitudinal context in SLE in one previous study, performed by Park *et al*.^37^. However, this study contained only 40 participants followed up over 4 weeks. Here, we show that elevated IL-10 levels are associated with subsequent disease activity. The ability for a clinician to anticipate which patients may require more aggressive management or more frequent monitoring would represent a significant breakthrough in the management of SLE. In light of this, the predictive capacity of IL-10 should be investigated further, and evaluated in combination with other predictive biomarkers to generate a potentially more powerful composite measure^42^.

We found serum IL-10 to be higher in the samples of patients taking glucocorticoids. Glucocorticoids have been shown to up-regulate the production of IL-10 *in vitro*^43^, but importantly we found that the associations of serum IL-10 with glucocorticoids was attenuated after adjustment for disease activity. Higher IL-10 concentrations were also seen in SLE patients of Asian ethnicity. While studies of IL-10 in SLE have been performed in cohorts with a range of ethnic predominance, the small sample sizes of these studies made it difficult to develop a clear pattern of IL-10 in different ethnic groups. It is not clear to what extent specific cytokines are involved in the causation of the more severe pattern of disease seen in Asian SLE patients^44^. The role that IL-10 might play in this phenotype, however, presents an interesting area for future research. Of note, ethnicity was not recorded for healthy controls used in this study, but the low rates of detection of IL-10 and IL-37 in controls suggest this was not a confounder of the difference between controls and SLE patients.

Our data also demonstrates significantly higher levels of serum IL-10 in patients with specific organ disease in SLE, including musculoskeletal activity, renal disease, serositis and serological activity. Similar differences were not seen between patients with and without CNS, vasculitis or cutaneous disease or in patients with or without fever or heamatoligical activity. However, with the exception of cutaneous and heamatological activities, numbers of patients in the affected groups were very low (1 or 3), so greater numbers are needed to fully assess these comparisons.

IL-37 is an anti-inflammatory cytokine expressed by a range of immune and non-immune cells^26^,^30^, but its function and role in disease is not well understood. IL-37 has, however, been suggested to be a cytokine biomarker in SLE, as it was previously found to be significantly higher in SLE patients compared to controls^31^. Here, we also found serum IL-37 to be higher in SLE patients, but in contrast to the previous study^31^, we saw no associations between serum IL-37 and disease activity. One possible reason for this discrepancy may be the differences in cohorts between these two studies. Song *et al*. studied only treatment-naïve Asian patients with highly active disease (SLEDAI-2k ≥10)^31^, whereas our cohort included patients from different ethnic backgrounds with a range of disease activity and treatments. Nonetheless, subgroup analysis, examining only Asian patients with highly active disease, failed to replicate the results of Song *et al*. (data not shown). It should be noted, however, that IL-37 was detected in fewer patients than IL-10, so a larger cohort might reveal more positive associations. It is not clear whether this is due to technical issues (i.e. sensitivity of the ELISA), or other reasons. Indeed, in many cases, associations with IL-37 show similar trends to IL-10, but would require more samples to achieve significance.

In conclusion, in the largest study to date supported by longitudinal data, we show a strong association of serum IL-10 with disease activity in SLE, wherein levels are higher in active compared to inactive disease, are higher in Asian patients and in association with renal disease, and are predictive of subsequent disease activity. Our data do not indicate a strong association of serum IL-37 with disease activity, although we suggest an even larger cohort is required to validate this.

## Methods

### Patients and clinical assessments

Between May 2007 and January 2012, all patients aged over 18 fulfilling the 1982 American College of Rheumatology (ACR) criteria^45^ who received care at the Monash Lupus Clinic, Melbourne Australia, were invited to participate in a longitudinal study of disease activity and biomarkers, as previously described^46^,^47^. Patients received standard-of-care therapy and were assessed at clinic visits as scheduled by the treating physician. Patients were included in the current study if complete clinical data and matching serum samples were available on up to three consecutive visits. Birth date, gender, ethnicity and year of disease onset were recorded at the first visit. Documentation of disease activity was performed using SLEDAI-2k^48^ at each clinic visit. Active disease was defined as a SLEDAI-2k score > 4 and highly active disease was defined as a score ≥ 10. Persistently active disease (PAD) was classified according to Nikpour *et al*., as ≥2 consecutive visits with a SLEDAI-2k 4, excluding SLEDAI-2k scores comprising exclusively serological parameters^49^. To account for disease activity over time, the time-adjusted mean SLEDAI (AMS) was derived^50^. In this cohort, disease-related organ damage is recorded annually using the Systemic Lupus International Collaborating Clinics (SLICC) SLE Damage Index (SDI)^51^. The presence of active SLE in specific organs was determined as described^46^,^47^, on the basis of a positive score in the relevant categories of the SLEDAI-2k. For example, active renal disease was defined as a non-zero score in any of the renal components of the SLEDAI-2k. Anti-dsDNA antibodies were measured using a Farr assay (Anti-dsDNA kit, Trinity Biotech, Wicklow, Ireland) with a normal value of <7 or ELISA with a normal value of <29 and are expressed as a ratio to the upper limit of normal in the assay used. Data are represented as fold increase over the normal value. Written informed consent was obtained from all subjects. All experimental protocols were approved by the Human Research Ethics Committee, Monash Health. All methods were carried out in accordance with the relevant guidelines.

### Serum cytokine quantification

Venous blood was collected in conjunction with routine laboratory testing for each visit, and serum obtained and stored at − 80 °C until analysis, as described^46^,^47^. Control serum was obtained from 28 healthy individuals whose age and gender was not significantly different to the patients. All controls had normal ESR, indicating that they represent a ‘non-inflamed’ population.

Specific enzyme-linked immunosorbent assay (ELISA) kits were used to measure serum IL-10 (Biolegend, San Diego, CA, USA) and IL-37 (Adipogen, Liestal, Switzerland) according the manufacturer’s protocols. Each sample was tested in duplicate. Where multiple samples over time were available, a TAM IL-10 or IL-37 concentration was calculated^46^. Any samples that exceeded the upper limit of the standard curve was subsequently diluted in PBS with 1% BSA and re-tested.

### Statistical analysis

All statistical analyses were performed using Stata version 13.1 (StataCorp, College Station, Texas, USA). For the samples which had readings below the detection limit of the ELISA (3.9 pg/mL for IL-10 and 16 pg/mL for IL-37), a value of 1 was assigned. Sensitivity analyses were performed by including and excluding the readings below the limit of detection, and the results were unchanged; therefore, the data including all samples are presented.

Continuous variables were described as median (interquartile range [IQR], range) and categorical variables were described as frequency (%). Comparison of quantitative values was performed using the Mann-Whitney U test for unpaired data. Spearman’s rank correlation was used to examine correlations between two variables.

Both IL-10 and IL-37 values were positively skewed, therefore, they were log_10_ transformed in order to carry out linear regression analyses to examine their associations with demographics and clinical phenotypes. The serum IL-10 and IL-37 levels have been reported as the geometric means in pg/mL with corresponding 95% confidence intervals. In addition, logistic regression analysis was performed to examine whether IL-10 measured at first visit was associated with subsequent disease activity. Variables with p-value < 0.1 in the univariable regression models were included in multivariable regression models. A *p* value < 0.05 was considered statistically significant.

## Additional Information

**How to cite this article**: Godsell, J. *et al*. Clinical associations of IL-10 and IL-37 in systemic lupus erythematosus. *Sci. Rep.* **6**, 34604; doi: 10.1038/srep34604 (2016).

## Supplementary Material

Supplementary Information

## Figures and Tables

**Table 1:** Characteristics of SLE study population.

	Total (N = 141)		
	Median	[IQR]	(Range)
Age at diagnosis	42	[32, 52]	(15, 80)
Disease duration (*months*)	103	[54.8, 199]	(18, 439)
Study period (*days*)	182	[119, 336]	(0, 1225)
Number of ACR criteria	5	[4, 6]	(4, 9)
SLICC-SDI (first visit)	0	[0, 1]	(0, 4)
SLICC-SDI (last visit)	1	[0, 2]	(0, 9)
SLEDAI-2k (first visit)	4	[2, 6]	(0, 18)
TAM-SLEDAI	4.03	[2.3, 6.0]	(0, 15)
	**n**	**(%)**	
Females	118	(84%)	
Ethnicity			
Caucasian	78	(55%)	
Asian	58	(41%)	
Other	5	(4%)	
Anti-nuclear antibodies >160^1^	136	(97%)	
Anti dsDNA positivity at first visit^1^	81	(57%)	
Anti dsDNA : upper limit ratio1,^2^			
1^st^ quartile (0, 0.43)	33	(24%)	
2^nd^ quartile (0.55, 1.29)	40	(29%)	
3^rd^ quartile (1.41, 3.83)	31	(22%)	
4^th^ quartile (4.14, 19.0)	36	(26%)	
Damage recorded at first visit(SDI > 0)	66	(47%)	
Active disease at first visit (SLEDAI-2k > 4)	55	(39%)	
Adjusted mean SLEDAI (AMS) > 4	68	(50%)	
Organ activity during study period			
CNS +ve	13	(9%)	
vasculitis +ve	9	(6%)	
MSK +ve	28	(20%)	
Renal +ve	31	(22%)	
Cutaneous +ve	53	(38%)	
Serositis +ve	6	(4%)	
Serological +ve	111	(79%)	
Fever +ve	7	(5%)	
Heam. +ve	15	(11%)	
Medications used during study period			
Prednisolone	96	(68%)	
Hydroxychloroquine	129	(91%)	
Methotrexate	17	(12%)	
Azathioprine	45	(32%)	
Mycophenolate Mofetil	27	(19%)	
Other^3^	11	(8%)	
Serum cytokines^4^	Median	[IQR]	(Range)
IL-10 (first visit) (*pg/mL*)	12.6	[1,43]	(1, 964)
TAM IL-10 (*pg/mL*)	14.2	[6.3, 51.7]	(1, 608)
IL-37 (first visit) (*pg/mL*)	48.1	[1, 271]	(1, 2365)
TAM IL-37 (*pg/mL*)	70.8	[8.9, 360]	(1, 2220)

IQR = Inter-quartile range; TAM = time adjusted mean.

^1^Data missing for 1 patient.

^2^dsDNA titres are reported as the ratio of the result to the upper limit of normal in the assay used.

^3^Other medications include cyclosporin, cyclophosphamide, leflunomide and rituximab.

^4^Any cytokine concentration below the detectable limit of the assay (3.9 or 16 pg/ml for IL-10 or IL-37, respectively) was recorded as 1 for analysis purposes.

**Table 2:** Factors associated with increased IL-10 expression measured at first visit.

	Serum levels of IL-10 derived from univariable linear regression analysis					Serum levels of IL-10 derived from multivariable linear regression analysis				
	GM	(95% CI)	Ratio of GM	(95% CI)	p-value	GM*	(95% CI)	Ratio of GM*	(95% CI)	p-value
Gender										
Females	11.8	(8.06, 17.3)	1.00							
Males	9.90	(4.17, 23.5)	0.84	(0.33, 2.16)	0.7					
Age groups										
<40	17.6	(10.3, 29.8)	1.00			14.3	(8.45, 24.2)	1.00		
40 to 59	8.12	(4.76, 13.8)	0.46	(0.22, 0.98)	0.04	9.15	(5.42, 15.4)	0.64	(0.30, 1.36)	0.2
≥60	9.03	(3.77, 21.6)	0.51	(0.18, 1.43)	0.2	11.5	(4.75, 28.0)	0.81	(0.28, 2.32)	0.7
Ethnicity										
non-Asians	8.30	(5.30, 13.0)	1.00			8.80	(5.62, 13.8)	1.00		
Asians	18.2	(10.7, 31.1)	2.20	(1.09, 4.42)	0.03	16.8	(9.73, 28.8)	1.90	(0.92, 3.94)	0.08
Prednisolone use reported at first visit										
no	6.14	(3.42, 11.0)	1.00			8.21	(4.48, 15.0)	1.00		
yes	15.8	(10.4, 24.1)	2.58	(1.25, 5.30)	0.01	13.6	(8.93, 20.8)	1.66	(0.77, 3.57)	0.19
Organ damage at first visit										
no	10.5	(6.53, 17.0)	1.00							
yes	12.6	(7.58, 21.0)	1.20	(0.59, 2.41)	0.6					
Disease activity at first visit										
SLEDAI-2k ≤ 4	7.51	(4.87, 11.6)	1.00			8.44	(5.42, 13.2)	1.00		
SLEDAI-2k > 4	22.2	(12.9, 38.2)	2.96	(1.48, 5.92)	0.002	18.5	(10.5, 32.6)	2.19	(1.04, 4.64)	0.04

GM = Geometric Mean; 95 CI = 95% Confidence Interval. *Ratios adjusted for age, ethnicity, prednisolone use and disease activity.

**Table 3:** Factors associated with IL-37 expression measured at first visit.

	Serum levels of IL-37 derived from univariable linear regression analysis					Serum levels of IL-37 derived from multivariable linear regression analysis				
	GM	(95% CI)	Ratio of GM	(95% CI)	p-value	GM*	(95% CI)	Ratio of GM*	(95% CI)	p-value
Gender										
Females	22.8	(13.6, 38.2)	1.00							
Males	9.48	(2.94, 30.6)	0.42	(0.12, 1.50)	0.18					
Age groups										
<40	37.2	(18.1, 76.3)	1.00			30.0	(14.7, 61.1)	1.00		
40 to 59	12.1	(5.85, 24.9)	0.32	(0.12, 0.90)	0.03	11.8	(5.8, 23.8)	0.39	(0.14, 1.07)	0.07
≥60	13.1	(4.01, 43.1)	0.35	(0.09, 1.42)	0.14	25.2	(7.4, 85.7)	0.84	(0.20, 3.59)	0.8
Ethnicity										
non-Asians	11.2	(6.14, 20.5)	1.00			11.7	(6.35, 21.5)	1.00		
Asians	44.4	(21.6, 91.4)	3.96	(1.55, 10.1)	0.004	41.9	(20.0, 87.5)	3.58	(1.34, 9.58)	0.01
PNL use reported at first visit										
no	13.5	(5.99, 30.4)	1.00							
yes	24.0	(13.4, 43.1)	1.78	(0.65, 4.84)	0.3					
Organ damage at first visit										
no	31.4	(16.5, 59.7)	1.00			29.6	(15.8, 55.7)	1.00		
yes	11.7	(5.88, 23.2)	0.37	(0.15, 0.95)	0.04	12.5	(6.4, 24.4)	0.42	(0.17, 1.07)	0.07
Disease activity at first visit										
SLEDAI-2k ≤ 4	18.5	(10.1, 34.0)	1.00							
SLEDAI-2k > 4	21.9	(10.2, 46.9)	1.18	(0.45, 3.14)	0.7					

GM = Geometric Mean; 95 CI = 95% Confidence Interval. *Ratios adjusted for age, ethnicity and organ damage.

**Table 4:** Multivariable associations of high active disease (AMS > 4).

	Low disease activity (AMS ≤ 4)^1^ N = 63 n (%)	High disease activity (AMS > 4)^1^ N = 60 n (%)	Adjusted odds ratio for subsequent high disease activity		
			Odds Ratio*	(95% CI)	p-value
Baseline IL-10					
1st quartile (1, 1)	28 (68%)	13 (32%)	1.00		
2nd quartile (3.99, 12.9)	12 (52%)	11 (48%)	2.14	(0.73, 6.33)	0.17
3rd quartile (13.0, 43.5)	12 (43%)	16 (57%)	2.61	(0.93, 7.28)	0.07
4th quartile (44.8, 964)	11 (35%)	20 (65%)	3.63	(1.31, 10.1)	0.01
Prednisolone Use ever					
No	28 (68%)	13 (32%)	1.00		
Yes	35 (43%)	47 (57%)	2.68	(1.17, 6.16)	0.02
Ethnicity					
Non-Asians	38 (56%)	30 (44%)	1.00		
Asians	25 (45%)	30 (55%)	1.06	(0.49, 2.32)	0.9

^1^Restricted to patients with two or more visits.

^*^Odds ratios adjusted for baseline IL-10, PNL use and ethnicity.
