# Supplementary material for: Clinical associations of IL-10 and IL-37 in systemic lupus erythematosus
Source: Sci Rep. 2016 Oct 6;6:34604. doi: 10.1038/srep34604 (PMC5052569; doi:10.1038/srep34604)
Supplement: Supplementary Information [file srep34604-s1.docx]

**Supplementary Information:**

**Clinical associations of IL-10 and IL-37 in systemic lupus erythematosus**

Jack Godsell^1§^, Ina Rudloff ^2,3§^, Rangi Kandane-Rathnayake^1^, Alberta Hoi ^1^, Marcel F. Nold ^2,3^, Eric F. Morand ^1^* & James Harris ^1^*

**SUPPLEMENTARY FIGURE LEGENDS**

**Supplementary Figure S1. Serum IL-37 is not associated with longitudinal assessments of disease activity in SLE.** (**A**) Linear regression analysis was used to examine a correlation between time-adjusted mean (TAM) serum IL-37 and time adjusted mean SLEDAI-2k (AMS). (**B**) comparison of TAM IL-37 in serum from patients with or without persistently active disease (PAD), measured by ELISA. (**C**) Linear regression analysis of baseline serum IL-37 with AMS. (**D**) comparison of baseline IL-37 in serum from patients with or without persistently active disease (PAD), measured by ELISA. Horizontal lines depict the median.
